# Non-linear relationship between TSH and psychotic symptoms on first episode and drug naïve major depressive disorder patients: a large sample sized cross-sectional study in China

**DOI:** 10.1186/s12888-024-05860-7

**Published:** 2024-06-04

**Authors:** Ruchang Yang, Zhe Li, Yingzhao Zhu, Yuxuan Wu, Xinchuan Lu, Xueli Zhao, Junjun Liu, Xiangdong Du, Xiangyang Zhang

**Affiliations:** 1 Suzhou Medical College of Soochow University, Suzhou, China; 2 Suzhou Guangji Hospital, The Affiliated Guangji Hospital of Soochow University, Suzhou, China; 3 Nanjing Meishan Hospital, Nanjing, China; 4 CAS Key Laboratory of Mental Health, Institute of Psychology, Chinese Academy of Sciences, Beijing, China

**Keywords:** Major depressive disorder, Thyroid-stimulating hormone(TSH), Psychotic symptom, First episode, Drug naive

## Abstract

**Introduction:**

Psychotic depression (PD) is characterized by the co-occurrence of emotional dysfunction and psychotic symptoms such as delusions and hallucinations with poor clinical outcomes. TSH may involve in the development of PD. This study aims to explore relationship between TSH and PD.

**Methods:**

A total of 1718 outpatients diagnosed as FEDN MDD were recruited in this study. The relationship between PD and TSH was evaluated using multivariable binary logistic regression analysis. To assess the presence of non-linear associations, a two-piecewise linear regression model was employed. Furthermore, interaction and stratified analyses were conducted with respect to sex, education, marital status, comorbid anxiety, and suicide attempt.

**Results:**

Multivariable logistic regression analysis revealed that TSH was positively associated with the risk of PD after adjusting for confounders (OR = 1.26, 95% CI: 1.11 to 1.43; *p* < 0.05). Smoothing plots showed a nonlinear relationship between TSH and PD, with the inflection point of TSH being 4.94 mIU/L. On the right of the inflection point, for each unit increase in serum TSH level on the right side of the inflection point, the probability of PD increased substantially by 47% (OR = 1.47, 95% CI: 1.25 to 1.73, *p* < 0.001), while no significant association was observed on the left side of the inflection point (OR = 0.87, 95% CI: 0.67 to 1.14, *p* = 0.32).

**Conclusion:**

Our investigation showed a nonlinear TSH-PD relationship in FEDN MDD patients, thus contributing to effective intervention strategies for psychotic symptoms in depression patients.

## Introduction

Major depressive disorder (MDD) ranks consistently among the top ten causes of years lived with disability (YLDs) in every country [1], and it is the most prevalent mood disorder in China [2]. Psychotic depression (PD) is characterized by the co-occurrence of emotional dysfunction and psychotic symptoms such as delusions and hallucinations [3]. Previous research has indicated a high prevalence of PD among patients with MDD, with rates ranging from 5.6 to 45% [4–7]. PD patients, in comparison to those with non-psychotic depression (NPD), demonstrate poorer clinical outcomes [8], including higher mortality rates [9–11] and increased incidence of suicide attempts [10].

Researchers suggested that the presence of psychotic symptomatology in depression is functionally and etiologically highly relevant [12]. Within this framework, thyroid hormones have emerged as key players in this complex relationship [13]. MDD patients with thyroid dysfunction comorbid hypothyroidism were more likely to present psychotic features and experience more severe depression [14]. Furthermore, a body of prior research has consistently reported associations between aberrations in the hypothalamus-pituitary-thyroid (HPT) axis and the manifestation of both psychotic symptoms [15–18] and depression [19–22].

Thyroid-stimulating hormone (TSH), synthesized in the pituitary gland, offers a more sensitive reflection of HPT axis function compared to other hormones [23]. For instance, in subclinical thyroid disorders, thyroid hormone levels are still in normal range, but with TSH rising or declining, we can predict potential disturbance of thyroid function and apply early intervention at subclinical stage for better clinical outcome. Though previous investigations have focused on relationship between psychotic symptoms and thyroid function, no consistent consensus has been reached thus far [15, 24–29]. Besides, a limited number of studies have focused on their relationship among MDD patients [30–32]. Consequently, this study delves into the specific association between TSH and PD. Furthermore, it is worth highlighting that our research exclusively recruited first-episode and drug naïve (FEDN) MDD patients, effectively minimizing the potential confounding effects of medication [33]. Moreover, considering the possibility of a non-linear relationship between TSH and PD, this study employs advanced statistical analyses to better elucidate their correlations.

## Methods

### Subjects

From 2015 to 2017, this cross-sectional study included 1718 outpatients from the First Hospital of Shanxi Medical University.

The study inclusion criteria were: (1) Han nationality; (2) age between 18 and 60 years; (3) a diagnosis of MDD based on DSM-IV conducted by two trained clinical psychiatrists; (4) depressive symptoms were first-episode without any prior antidepressant, antipsychotic; (5) a duration of illness no longer than 24 months; (6) a minimum score of 24 on the 17-item Hamilton Rating Scale for Depression (HAMD-17); (7) no previous thyroxine therapy, or any specific medications. Exclusion criteria were: (1) having a severe physical disease, such as organic brain diseases or severe infection; (2) presence of any other major SCID-based DSM-IV Axis I disorder; (3) pregnancy or lactation; (4) alcohol or substance dependence or abuse except for tobacco smoking [34].

All participants voluntarily agreed to participate in the study and provided written informed consent prior to enrollment. The study protocol was approved by the medical ethics committee at the First Hospital of Shanxi Medical University.

### Socio-demographic characteristics

Well-trained researchers collected a range of socio-demographic characteristics from the participants, including age, gender, age at onset, illness duration, marital status, education level, systolic blood pressure (SBP), diastolic blood pressure (DBP), and body mass index (BMI).

### Clinical measures

The assessment of depression severity in this study was conducted using the 17-item Hamilton Depression Scale (HAMD), while anxiety severity was measured using the 14-item Hamilton Anxiety Rating Scale (HAMA). To ensure unbiased data collection, two qualified psychiatrists, unaware of participants’ clinical conditions, collected the aforementioned information through the Structured Clinical Interview for DSM Disorders (SCID). The inter-rater reliability of the HAMD, HAMA, and PANSS-P total scores was assessed through repetitive evaluations, resulting in observer correlation coefficients exceeding 0.8. Consistent with previous studies [35, 36], a cutoff point 15 for PANSS-P was used to identify PD in FEDN MDD patients. Suicide attempt, which was defined as self-injurious behavior, with an intention to end one’s life, but not resulting in death, was evaluated through a face-to-face interview with subjects and/or their family members by question “Have you (he or she) ever attempted suicide in your (or the patient’s) lifetime?” Interviewees responded “yes” to this inquiry were regarded as suicide attempters.

### Blood sample

Following an overnight fast, blood samples were collected from the participants between 6:00 and 8:00 a.m. and promptly sent for testing before 11 a.m. The measurement of serum TSH levels was performed by the hospital laboratory center using the Roche C6000 Electrochemiluminescence Immunoassay Analyzer (Roche Diagnostics, Indianapolis, IN, USA). The established normal range for TSH was 0.27–4.20 mIU/L. Additionally, various other blood biomarkers, including free triiodothyronine (FT3), free thyroxine (FT4), thyroid peroxidase antibody (TPOAb), anti-thyroglobulin (TgAb), high-density lipoprotein cholesterol (HDL-C), and low-density lipoprotein cholesterol (LDL-C), were all measured on the same day. To facilitate analysis, the patients were divided into three groups of equal size based on their serum TSH levels, namely low, middle, and high groups [34].

### Statistical analysis

Continuous variables were presented according to their distribution. Non-normally distributed data were reported as the median with interquartile range (IQR), while normally distributed variables were expressed as the mean with standard deviation (SD). Categorical variables were presented as frequencies and percentages. To assess differences between the tertile groups of serum TSH levels, a one-way ANOVA test, Kruskal-Wallis H test, or χ2 test was employed. The linear relationship between the serum TSH levels and psychotic symptoms was estimated using logistic regression models, and the serum TSH level was examined as both continuous and categorical variables based on tertiles. Unadjusted and adjusted odds ratios (ORs) were presented with 95% confidence intervals (CIs). Sensitivity analyses were conducted to ensure the robustness of the data analysis. In order to determine a *p*-value for trend, the serum TSH level was transformed into a categorical variable. The variance inflation factor (VIF) was used to assess multicollinearity among independent variables, with covariates having VIF values above 5.0 excluded from the final model. Potential confounders were selected if they changed the estimates of serum TSH level on PD by more than 10% or had a *p*-value of less than 0.10 in univariable analysis. Three models were constructed to verify the stability of the results: an unadjusted model, Model I adjusted for sex and age, and Model II adjusted for age, gender, education, HAMA, HAMD, TGAb, TPOAb, TC, TG, FBG, HDL-c, LDL-c, SBP, and DBP. The non-linear relationship between serum TSH levels and PD was assessed using smoothing plots, and the threshold impact suggested by the smoothing plot was investigated using a two-piecewise linear regression model based on the generalized estimating equation (GEE) and an inflection point was calculated using a recursive algorithm. Stratified analyses were performed based on sex, education, marital status, comorbid anxiety, and suicide attempt. The interaction effects within various subgroups variables were evaluated using the log-likelihood ratio test. All statistical analyses were performed using the R software packages (http://www.r-project.org, The R Foundation) and EmpowerStats (http://www.empowerstats.com, X&Y Solution, Inc., Boston, Massachusetts, United States). GraphPad Prism 8.0 was used to create the visualizations. Statistical significance was defined as two tails of *p* < 0.05.

## Results

### Baseline characteristics

**Fig. 1:**
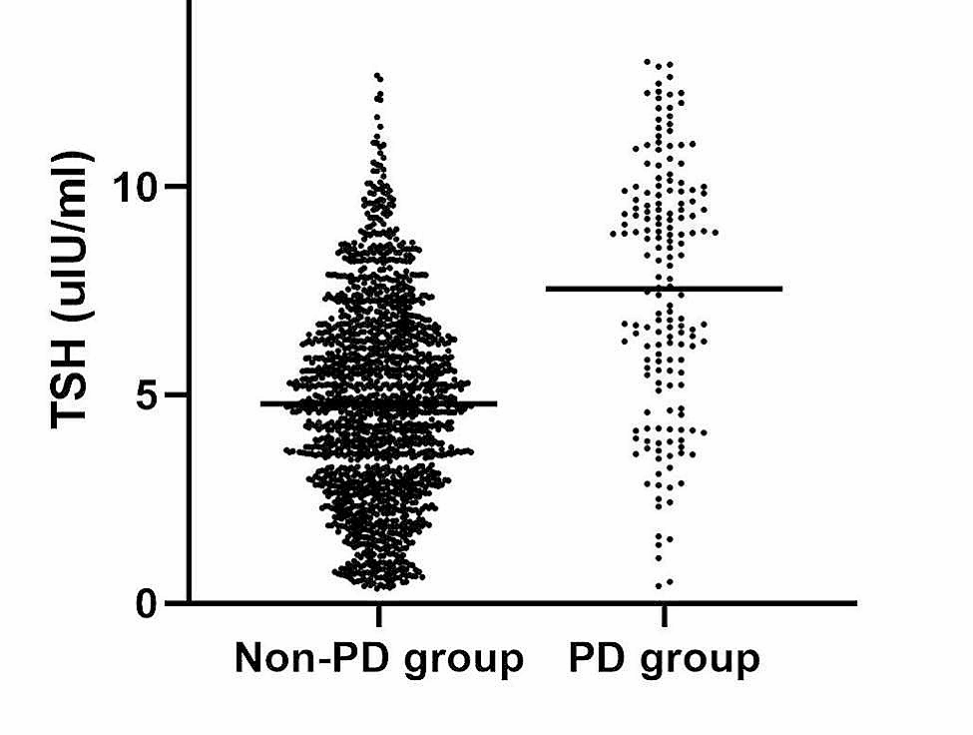
Distribution of TSH in FEDN MDD patients with or without psychotic symptoms

Table 1 lists the participant characteristics categorized based on tertiiles of serum TSH level. Significant correlations between serum TSH level tertiles and several variables, including age, duration of illness, age at onset, HAMD, HAMA, TGAb, TPOAb, FBG, TC, TG, HDL-c, LDL-c, BMI, SBP, DBP, comorbid anxiety, psychotic symptoms and suicide attempt (all *p* < 0.05), were found. Figure 1 demonstrates the distribution of TSH in FEDN MDD patients with or without psychotic symptoms.

**Table 1:** Socio-demographical and clinical characteristics of the participants

Variables	TSH (uIU/ml) tertile			*P*-value
	Low (0.36–3.78)	Middle (3.78–6.05)	High (6.06–13.01)	
*N*	571	574	573	
Age (years)	33.91 ± 12.24	34.56 ± 12.42	36.14 ± 12.54	< 0.05
Duration of illness (months)	4.98 ± 3.85	7.11 ± 5.08	7.08 ± 4.71	< 0.001
Age at onset (years)	33.78 ± 12.20	34.31 ± 12.27	35.89 ± 12.38	< 0.05
HAMD	28.60 ± 2.61	30.26 ± 2.60	32.02 ± 2.56	< 0.001
HAMA	19.98 ± 2.81	19.89 ± 3.32	22.53 ± 3.57	< 0.001
TGAb (IU/l)	41.53 ± 88.54	85.46 ± 205.28	142.92 ± 339.60	< 0.001
TPOAb (IU/l)	35.22 ± 98.36	58.86 ± 120.46	122.62 ± 228.56	< 0.001
FT3 (pmol/l)	4.87 ± 0.68	4.93 ± 0.76	4.92 ± 0.72	0.285
FT4 (pmol/l)	16.79 ± 3.09	16.52 ± 3.09	16.80 ± 3.10	0.240
FBG (mmol/l)	5.09 ± 0.56	5.38 ± 0.52	5.73 ± 0.68	< 0.001
TC (mmol/l)	4.64 ± 0.93	5.03 ± 0.84	6.09 ± 0.98	< 0.001
HDL-c (mmol/l)	1.31 ± 0.24	1.30 ± 0.25	1.05 ± 0.29	< 0.001
TG (mmol/l)	2.05 ± 1.01	2.11 ± 0.93	2.35 ± 0.99	< 0.001
LDL-c (mmol/l)	2.64 ± 0.73	2.80 ± 0.75	3.51 ± 0.83	< 0.001
BMI (kg/m2)	23.84 ± 1.73	24.65 ± 1.90	24.61 ± 2.02	< 0.001
Systolic pressure (mmHg)	113.4 ± 10.73	118.85 ± 8.99	126.11 ± 9.02	< 0.001
Diastolic pressure (mmHg)	73.51 ± 6.58	75.37 ± 5.95	78.96 ± 6.52	< 0.001
Gender				0.998
Male	195 (34.15%)	197 (34.32%)	196 (34.21%)	
Female	376 (65.85%)	377 (65.68%)	377 (65.79%)	
Education				0.112
Junior high school	115 (20.14%)	145 (25.26%)	153 (26.70%)	
Senior high school	264 (46.23%)	256 (44.60%)	240 (41.88%)	
College	162 (28.37%)	145 (25.26%)	142 (24.78%)	
Postgraduate	30 (5.25%)	28 (4.88%)	38 (6.63%)	
Marital status				0.217
Single	173 (30.30%)	177 (30.84%)	152 (26.53%)	
Marriage	398 (69.70%)	397 (69.16%)	421 (73.47%)	
Suicide attempt				< 0.001
No	502 (87.92%)	515 (89.72%)	355 (61.95%)	
Yes	69 (12.08%)	59 (10.28%)	218 (38.05%)	
comorbid anxiety				< 0.001
No	138 (24.17%)	154 (26.83%)	46 (8.03%)	
Yes	433 (75.83%)	420 (73.17%)	527 (91.97%)	
Psychotic symptoms				< 0.001
No	548 (95.97%)	544 (94.77%)	455 (79.41%)	
Yes	23 (4.03%)	30 (5.23%)	118 (20.59%)	

The variables are presented as n (%) or the mean ± SD or median, HAMD, 17-item Hamilton Rating Scale for Depression; HAMA, 14-item Hamilton Anxiety Rating Scale; TSH, thyroid-stimulating hormone; TGAb, thyroglobulin antibody; TPOAb, thyroid peroxidase antibody; FT3, free triiodothyronine; FT4, free thyroxine; FBG, fasting blood glucose; TC, total cholesterol; HDL-c, high-density lipoprotein cholesterol; TG, triglyceride; LDL-c, low-density lipoprotein cholesterol; BMI, body mass index

### Associations between serum TSH level and PD

In the fully adjusted analysis (Table 2), a higher serum TSH level was substantially linked to a higher risk of PD, according to the fully adjusted data (OR = 1.26, 95% CI: 1.11 to 1.43; *p* < 0.05).

**Table 2:** Relationship between TSH and psychotic symptoms in different models

Variables	Non-adjusted		Adjust I		Adjust II	
	OR (95%CI)	*p*-value	OR (95%CI)	*p*-value	OR (95%CI)	*p*-value
TSH (uIU/ml)	1.52 (1.42, 1.63)	< 0.001	1.52 (1.42, 1.63)	< 0.001	1.26 (1.11, 1.43)	< 0.001
TSH (uIU/ml) tertile						
Low	Reference		Reference		Reference	
Middle	1.31 (0.75, 2.29)	0.336	1.31 (0.75, 2.28)	0.347	0.60 (0.29, 1.22)	0.157
High	6.18 (3.89, 9.82)	< 0.001	6.10 (3.83, 9.72)	< 0.001	1.73 (0.79, 3.76)	0.170
p for trend	< 0.001		< 0.001		0.227	

CI, confidence interval; unadjusted model adjusted for none; Model I adjusted for age, sex; Model II adjusted for age, gender, education, HAMA, HAMD, TGAb, TPOAb, TC, TG, FBG, HDL-c, LDL-c, SBP, and DBP.

**Fig. 2:**
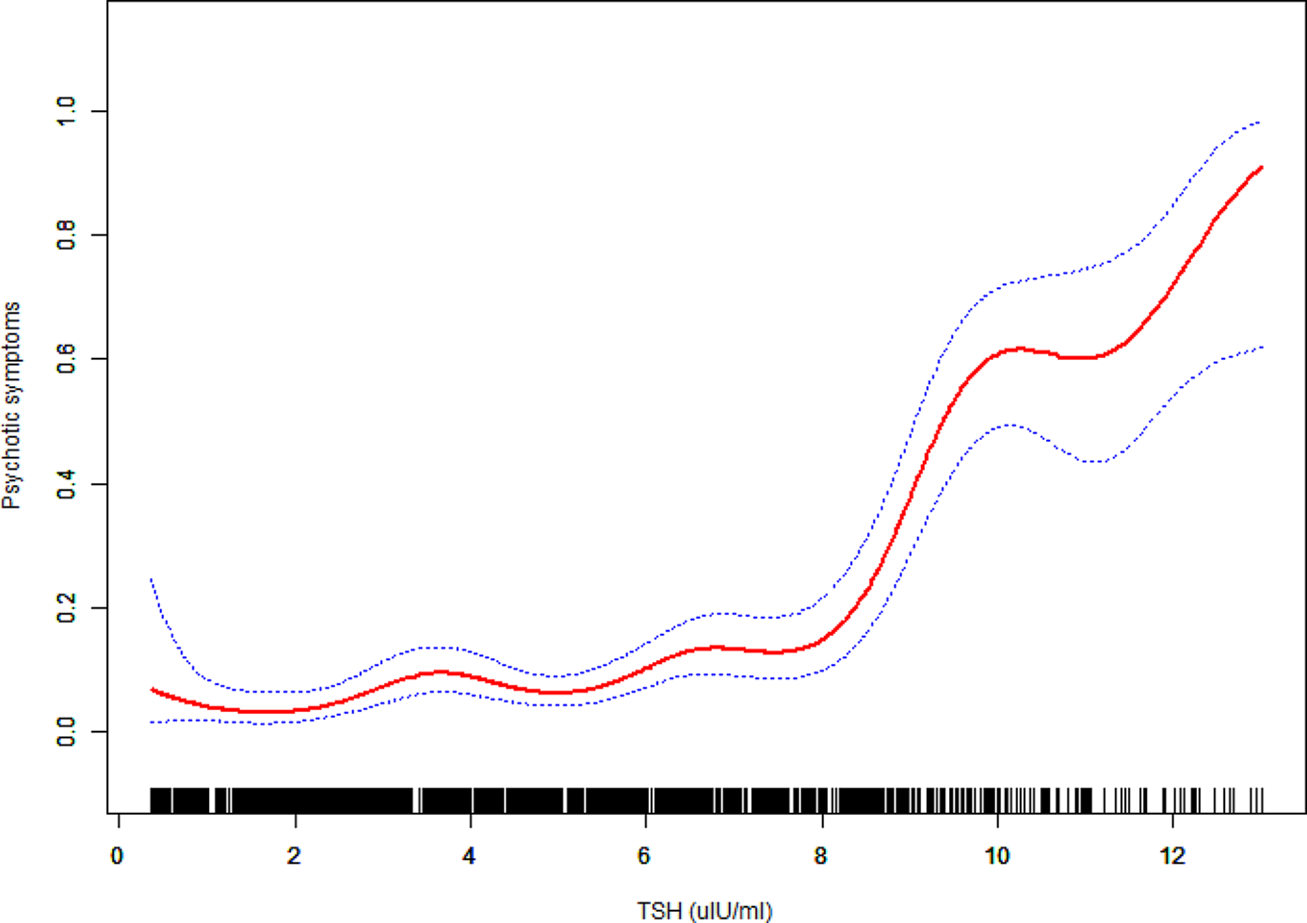
The relationship between TSH and the probability of psychotic symptoms. A nonlinear relationship between TSH and the probability of psychotic symptoms was observed after adjusting for age, gender, education, HAMA, HAMD, TGAb, TPOAb, TC, TG, FBG, HDL-c, LDL-c, SBP, and DBP

To further explore the relationship between serum TSH level and PD, generalized additive models were employed and depicted in Fig. 2. The analysis revealed a non-linear association between serum TSH level and PD, with a significant *p*-value for non-linearity (< 0.05). Subsequently, a two-segment logistic regression model was utilized to identify an inflection point at a serum TSH level of 4.94 mIU/L.

The results from the generalized additive models, as shown in Fig. 2, demonstrated a non-linear pattern between serum TSH level and PD, with a significant *p*-value for non-linearity (< 0.05). The two-segment logistic regression model indicated that for each unit increase in serum TSH level on the right side of the inflection point, the probability of PD increased substantially by 47% (OR = 1.47, 95% CI: 1.25 to 1.73, *p* < 0.001). However, on the left side of the inflection point, there was no significant evidence of a relationship between serum TSH level and PD (OR = 0.87, 95% CI: 0.67 to 1.14, *p* = 0.32), as presented in Table 3. Specifically, among the study participants, 842 individuals had a serum TSH level equal to or greater than 4.94 mIU/L, while 876 individuals had a serum TSH level less than 4.94 mIU/L.

**Table 3:** The results of two-piecewise logistic regression model

Inflection point of TSH	OR	95% CI	*p*-value
Inflection point	4.94		
< 4.94 slope	0.87	(0.67, 1.14)	0.324
$\ge$ 4.94 slope	1.47	(1.25, 1.73)	< 0.001
Slope2 - slope1	1.68	(1.20, 2.36)	< 0.05
Predicted at 4.94	-3.16	(-3.49, -2.83)	
Log likelihood ratio test			< 0.05

Effect: psychotic symptoms; cause: TSH; adjusted for age, gender, education, HAMA, HAMD, TGAb, TPOAb, TC, TG, FBG, HDL-c, LDL-c, SBP, and DBP. Slope 1 and slope 2 are the slope coefficients for the segment before and after the inflection point

### Subgroup analyses

**Fig. 3:**
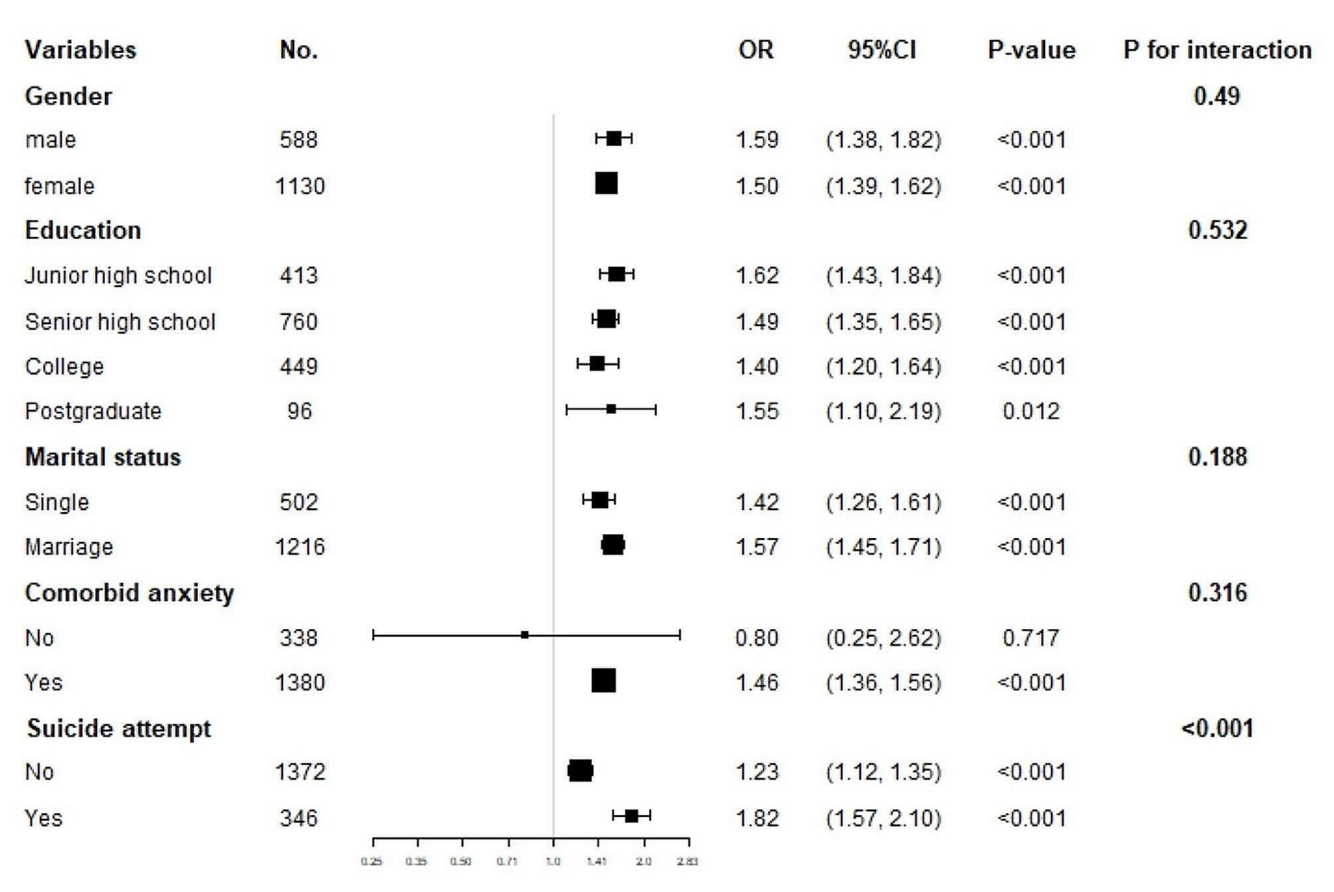
Subgroup analysis of the association between TSH and psychotic symptoms. The OR (95% CI) was derived from the Logistic regression model. (Age, sex, education, duration of illness, HAMD, HAMA, A-TG, A-TPO, TC, HDL-c, LDL-c, SBP and DBP were adjusted)

Figure 3 presents the results of the subgroup analysis, demonstrating consistent patterns across various subgroups, including sex (male, female), marital status (single, married), education level (junior high school, senior high school, college, postgraduate), and comorbid anxiety status (no, yes). No significant interaction effects were observed in any of these subgroups. However, in relation to suicide attempts, a statistically significant interaction effect was detected (*p* < 0.001).

## Discussion

In this population-based cross-sectional study, the association between serum TSH level and PD was examined after adjusting for covariables. The findings revealed a significant association, indicating that higher serum TSH levels were linked to PD. Furthermore, a non-linear correlation was observed, with an inflection point identified at 4.94 mIU/L. These results remained consistent across various subgroups, including gender, education, marital status, and comorbid anxiety. However, when considering the presence or absence of a suicide attempt, an interaction effect was observed. This highlights the need for further investigation into the associations between TSH levels and suicide attempts in PD patients.

The primary focus of this study was to investigate the relationship between serum TSH levels and PD in FEDN MDD patients. In the treatment of MDD, antidepressant drugs such as selective serotonin reuptake inhibitors (SSRIs), tricyclic antidepressants, serotonin-norepinephrine reuptake inhibitors, and monoamine oxidase inhibitors are commonly utilized [37]. Additionally, for cases of treatment-resistant depression, augmentation with second-generation antipsychotics and lithium has shown efficacy [38, 39]. Previous research has reported the impact of antidepressant drugs on thyroid function [40–46]. Consequently, it is plausible that the correlation between TSH levels and PD among FEDN MDD patients may exhibit distinct features compared to patients who have undergone drug treatment.

In our study, we observed a significant association between higher serum TSH levels and PD. This finding is consistent with several previous studies that have also reported a similar conclusion, suggesting that hypothyroidism characterized by elevated serum TSH levels and reduced serum T3 and T4 levels may contribute to the development of psychotic symptoms [24, 25, 47]. Thyroid hormones not only play a significant role in the development of cerebral cortex [48], but also exert an influence on regional cerebral glucose metabolism [49], the glioendocrine system [50], and the catecholaminergic and serotonin (5-HT) systems within the brain [51], collectively impacting an individual’s mental state. Deviation from normal thyroid hormone levels may induce variability in cerebral functions, thereby precipitating manifestations of psychosis. Moreover, thyroid dysfunction observed in PD patients may stem from an augmented release of cortisol triggered by an overactive hypothalamic-pituitary-adrenal (HPA) axis [52]. To further investigate the relationship between TSH and PD, we explored the correlation in a non-linear manner and identified an inflection point at 4.94 mIU/L. Below this threshold, there was no significant evidence of a relationship between serum TSH levels and PD. However, when TSH levels exceeded 4.94 mIU/L, the probability of PD substantially increased by 47% for each unit increase in serum TSH level. It is important to note that the normal range for TSH in our study was defined as 0.27–4.20 mIU/L, and the inflection point of 4.94 mIU/L was 0.74 mIU/L above the upper limit of the normal range. The fact that TSH levels below 4.94 mIU/L, although fluctuating, remained within or close to the normal range can be attributed to the regulatory capacity of our body. Even when certain hormones occasionally deviate from the normal range, our physiological and mental state tends to maintain balance and not exhibit abnormalities. However, when these deviations exceed a certain threshold, abnormalities may manifest and worsen. In our study, although the recommended upper limit of TSH was 4.2 mIU/L, as long as the TSH level remained below 4.94 mIU/L, there was no statistically significant association between TSH and PD. Conversely, when TSH levels exceeded 0.74 mIU/L beyond the upper limit of the normal range, a positive correlation with PD became apparent.

HPT axis operates through a negative feedback regulation mechanism. TSH does not directly affect somatic cells but instead modulates the concentration of thyroid hormones, which act directly on thyroid receptors in the nuclei of target cells, thereby exerting their biological effects. Changes in absolute or relative thyroid hormone concentrations can influence the secretion of thyrotropin-releasing hormone (TRH) in the hypothalamus, thereby regulating the overall HPT axis. Elevated serum TSH levels typically indicate a deficiency in absolute or relative thyroid hormone levels, such as reduced receptor sensitivity. A multicenter European study elucidated that MDD patients comorbid hypothyroidism – characterized as high TSH and low thyroid hormone levels - were more likely to exhibit psychotic features [14]. Fluctuations in thyroid hormone levels have been shown to impact various aspects of brain function, including regional cerebral glucose metabolism [49], the glioendocrine system [50], catecholaminergic system, and the brain serotonin (5-HT) system [51]. These alterations in brain cell function may contribute to the development of depression and the manifestation of psychotic symptoms.

Alternatively, in cases where abnormalities arise within the central endocrine system, serum levels of TSH may exhibit abnormal elevations irrespective of thyroid hormone concentrations. TSH, being directly regulated by TRH, is subject to fluctuations if any disruptions occur in TRH secretion. Additionally, certain pituitary tumors can contribute to excessive TSH secretion, further exacerbating the abnormal elevation of TSH levels. Consequently, the levels of thyroid hormones also increase due to the heightened TSH activity. Previous investigations have established links between thyroid hormone levels and the manifestation of psychotic symptoms [53–56]. Duval et al. postulated that thyroid dysfunction observed in patients with psychotic disorders may stem from heightened cortisol secretion triggered by an overactive hypothalamic-pituitary-adrenal axis [52]. .

Several limitations should be noted. Firstly, due to its cross-sectional design, the ability to establish causal relationships between TSH and PD is inherently limited. Secondly, variations in TSH testing methods across different hospitals have resulted in the adoption of diverse reference standards. Consequently, caution must be exercised when interpreting the inflection point at 4.94 mIU/L. Thirdly, medication history was obtained through interviews with patients and their family members rather than relying on medical records. This introduces the potential for recall bias and may impact the accuracy of the data. Fourthly, all MDD patients were recruited from the outpatient department of a general hospital in Shanxi Province, China. Therefore, our findings should be extended cautiously to inpatients, community patients, and outpatients from other regions or racial groups. Fifthly, several confounding factors crucial to the study were not collected, such as employment status, family income, and serum TSH level before the onset of MDD. Future studies should impose repetitive examinations of TSH for a better exploration of the relationship between TSH and PD. Furthermore, the analysis of additional thyroid function indicators, including T3, T4, and TRH, could provide further insights into the impact of thyroid function on the development of PD.

## Conclusion

Our investigation showed a nonlinear TSH-PD relationship in FEDN MDD patients but differing TSH inflection points between comorbid suicide attempt, thus contributing to effective intervention strategies for psychotic symptoms in depression patients. Notably, we observed a noteworthy positive correlation between serum TSH levels and psychotic symptoms solely when TSH concentrations exceeded the threshold of 4.94 mIU/L.

## Data Availability

The data are available from the corresponding author on reasonable request.
